# Enhancement of Tight Junctional Barrier Function by Micronutrients: Compound-Specific Effects on Permeability and Claudin Composition

**DOI:** 10.1371/journal.pone.0078775

**Published:** 2013-11-13

**Authors:** Joanna Mercado, Mary Carmen Valenzano, Cameron Jeffers, Jason Sedlak, Marina K. Cugliari, Eleni Papanikolaou, Jacob Clouse, Jingya Miao, Nina E. Wertan, James M. Mullin

**Affiliations:** 1 Lankenau Institute for Medical Research, Wynnewood, Pennsylvania, United States of America; 2 Drexel University, Philadelphia, Pennsylvania, United States of America; Emory University School of Medicine, United States of America

## Abstract

Amid an increasing number of reports in the literature concerning epithelial barrier enhancement by various nutrient compounds, there has never been a study performing side-by-side comparisons of these agents in a single epithelial model. We compare five nutrient compounds (previously reported in various epithelial models to enhance barrier function) regarding their ability to increase transepithelial electrical resistance (R_t_) and decrease transepithelial mannitol permeability (J_m_) across LLC-PK_1_ renal epithelial cell layers. The effects of these nutrients on the abundance of various tight junctional proteins are also compared. In the overall group of nutrients tested - zinc, indole, quercetin, butyrate and nicotine - only nicotine failed to improve barrier function by either parameter. Nicotine also was without effect on tight junctional proteins. Quercetin simultaneously increased R_t_ and decreased J_m_. Zinc, butyrate and indole only exhibited statistically significant enhancement of R_t_. Each of these four effective nutrient compounds had unique patterns of effects on the panel of tight junctional proteins studied. No two compounds produced the same pattern of effects. This unique pattern of effects on tight junctional complex composition by each compound establishes the chance for additive or even synergistic improvement of barrier function by combinations of compounds. A synergistic effect of the combination of quercetin and zinc on R_t_ is shown.

## Introduction

The link between compromise of epithelial/endothelial barriers and a wide, diverse range of human diseases (cancer, inflammation, diabetes, multi-organ failure, etc.) has in some cases been known for many years, but is receiving increased interest as the understanding of epithelial/endothelial barriers has progressed. In just the last two years, numerous reviews have been written on this topic in addition to the large number of individual research papers on specific aspects of the phenomenon [1], [2], [3], [4], [5], [6], [7], [8]. Clearly the concept that breakdown of epithelial/endothelial tissue compartmentation – and specifically aberration of the tight junctional (TJ) complex - is integral to a wide array of morbidity, is now becoming firmly rooted in medical thinking.

After the initial identification in 1963 of the TJ as the element principally responsible for epithelial barrier function [9], it would take nearly 30 years of research by a multitude of laboratories for the concept to become fully appreciated that this structure is not static, but rather its permeability and composition are under dynamic regulation. Disease simply ‘hijacks’ those regulatory pathways toward its own end. But a fascinating and highly promising line of research began to gain traction within the last ten years, showing that various dietary components can exert regulatory effects of their own on TJs, and that these actions were often beneficial in leading to a less leaky barrier. A growing number of reviews have already been authored on this subject as well [10], [11], [12], [13].

Among the specific nutrients displaying barrier enhancement activity, are zinc, butyrate, quercetin, nicotine and indole, a decidedly ‘mixed bag’ regarding their chemical class and source in the diet. The history of zinc regarding TJ barriers appears to stem from observations of the correlation of zinc deficiency and pediatric diarrhea [14]. Zinc deficiency has been observed to compromise barrier function of CACO-2 cell layers [15]. Zinc supplementation, at high µM concentrations, improves barrier function with very specific effects on TJ complexes [16], [17]. Zinc supplementation can also offset the effects of conditions that themselves impair barrier function [18], [19], [20]. The short chain fatty acid, butyrate, at mM concentrations, also enhances epithelial barrier function, and also with specific modifications of the TJ complex [21], [22]. Whereas butyrate can be derived from gastrointestinal bacterial flora metabolism of dietary fiber, another bacterial metabolite, indole, derives from dietary tryptophan, and also has epithelial barrier enhancing properties. Nicotine, at µM levels, improved CACO-2 barrier function regarding electrical resistance and fluorescein permeability [23]. Indole, at mM levels, has been reported to not only increase transepithelial electrical resistance of a gastrointestinal cell line model, but to decrease epithelial production of proinflammatory cytokines that may negatively impact barrier function [24]. The bioflavonoid, quercetin, a component of green and black teas, also has properties of epithelial barrier improvement at µM concentrations, as do certain other compounds of the bioflavonoid class [12]. As is true of the other dietary components listed above, quercetin enhancement of epithelial barrier function appears to occur with structural/compositional modifications of the TJ complex [25], [26], [27].

These various agents had never been studied in a single epithelial model at the same time. In this current study, we sought to address one question – in a single epithelial model, do these various agents have distinct, unique effects on the tight junctional complex, and exert different permeability effects on the barrier, within their overall theme of barrier enhancement. Our focus was singularly on the actions *per se*, rather than the mechanism of action. Our purpose in this approach was to begin to lay groundwork for optimal therapeutic effects. The possibility that these compounds each enhance epithelial barriers by distinct actions upon different target proteins in the TJ complex, would create a scenario wherein combinations of these various nutrient compounds could in fact have additive or even synergistic effects in therapeutic settings. As we have shown in a recent study, these actions would be highly dependent upon the specific epithelial model under study [16], implying that optimal combinations would need to be worked out for each epithelial tissue.

## Materials and Methods

### Cell Culture

The LLC-PK_1_ culture, an epithelial cell line derived from the outer cortex of 141 porcine kidney, was a gift from Dr. Robert Hull (Eli Lilly) [28] and was used between passages 186 to 200. Upon confluence, cells were passaged on a weekly basis by trypsinizination (0.25% trypsin and 2.2 mM EDTA) and were seeded at 1×10^5^ cells/Falcon 75-cm^2^ culture flask with 25 ml of Eagle's minimum essential media, alpha-modified without nucleosides or ribonucleosides (Cellgro). The media was supplemented with 2 mM L-Glutamine (Cellgro) and 10% defined fetal bovine serum (HyClone). Cultures were incubated at 37°C in 95% air-5% CO_2_ atmosphere.

### Media Supplementation with Nutraceuticals

For each nutraceutical, with the exception of quercetin, a stock solution was prepared. A serial dilution was then done in culture medium to attain desired working concentrations for treatment of cell layers. Prior to supplementation, the media was filter sterilized with a 0.2 µm sterile syringe (Corning). For zinc, a stock solution (100 mM) was made from zinc sulfate heptahydrate (Fisher Chemical) in deionized distilled water. A butyrate stock solution (400 mM) was made from sodium butyrate (Sigma-Aldrich) in deionized distilled water. A 50 mM stock solution of nicotine (Sigma-Aldrich) was also made in deionized distilled water. In the case of indole (Sigma-Aldrich), a 400 mM stock solution was prepared in absolute ethanol. With quercetin (Sigma-Aldrich), dry chemical was added directly to complete culture medium to make up a working concentration (400 µM) that was applied directly to cells. (Solubilization of quercetin in medium at 400 µM required warming medium to 38°C for 40 minutes with constant stirring). Lower concentrations were prepared simply by serial dilution in complete medium. Proper solvent controls were performed in all experiments.

### Transepithelial Electrophysiology and Permeability

Cells were seeded into sterile Millipore Millicell polycarbonate (PCF) permeable supports (30 mm diameter with 0.4 µm pore size) on day 0 at a seeding density of 1×10^6^ cells/insert. Three sterile Millicell PCF inserts were placed into a 100 mm petri dish. On day 1, all cell layers were refed (both compartments) with control medium. On day 2 or 3 (depending upon the specific nutraceutical), cells were supplemented with their appropriate mediums (2 ml apical/15 ml basalateral) for either a 24 or 48 hour treatment (depending upon agent). Experiments were conducted on day 4.

On the day of transepithelial experiments, the cell layers were re-fed with fresh control medium and allowed to incubate at 37°C for 1.5 to 2 hours prior to electrophysiological readings. Potential difference (PD), transepithelial electrical resistance (R_t_), and short-circuit current (I_scc_) were measured using 1 sec, 40 µamp direct current pulses, and calculated using Ohm's law. As soon as electrical measurements were completed, the basal-lateral medium from the dish was aspirated and replaced with 15 ml of medium containing 0.1 mM, 0.25 µCi/ml ^14^C-D-mannitol (Perkin-Elmer, Boston, MA) and incubated at 37°C. Triplicate basal-lateral medium samples (50 µl) were taken for liquid scintillation counting (LSC) for specific activity determination. Duplicate samples (200 µl) were taken from the apical side at 45, 90, and 135 min for LSC to determine flux rates. The media lost due to sampling from the apical compartment was replaced with fresh medium of the same sample volume. The flux rate (in cpm/min/cm^2^ and pmol/min/cm^2^) was calculated for the ^14^C-D-mannitol diffusing across the cell layer.

### Analyses of Tight Junctional Proteins

Cells in culture flasks were allowed to grow to confluence, and then were re-fed with media containing the various nutraceuticals under study at the concentrations that provided maximal barrier enhancement, and for the same time periods used in the permeability studies. After the 24 or 48 hr incubations, cell layers were washed 2× in 4°C phosphate-buffered saline (PBS) and then harvested by physical scraping into lysis buffer, followed by sonication and ultra centrifugation. Samples of these fractions were analyzed by PAGE using a 4–20% gradient Novex Tris-glycine gel t 125 V for 1 hr 45 min. (8% Tris-glycine gels were used in the cases of occludin and tricellulin). Precision Plus Kaleidoscope Protein Standards methods were also included in each gel. Proteins were transferred at 30 V for 2 hr from the gel to a PVDF membrane. The membranes were then washed three times with PBS-T (0.3% Tween-20) for 10 min each and blocked with 5% milk/PBS-T for 1 hr at RT. Membranes were incubated with the specific primary antibody (anti-claudin-1, -2, -4, -5, anti-occludin, anti-tricellulin [Life Technologies]) at 1.0 µg/ml in 5% milk/PBS-T overnight at 4°C then 2 hr at RT. (For occludin, tricellulin and claudins -3 and -7, there was only a 2 minute incubation with the primary antibody at room temperature). The membranes were washed with PBS-T 3× for 10 minutes each, then incubated with secondary antibody (rabbit anti-mouse or goat anti-rabbit IgG labeled with horseradish peroxidase (Southern Biotechnology) for 1 hr at RT. Membranes were washed with PBS-T (4× for 10 minutes each), then treated for 1 min with Western Lighting-ECL chemiluminescence reagents. The membranes were then exposed to HyBlot CL autoradiography film (Denville Scientific) which was developed in a Kodak M35A X-OMAT processor. Band densities were quantified by densitometry. Band densities of nutraceutical-treated cell samples were compared against normalized averages of corresponding control band densities.

#### Statistics

For electrophysiology, radiotracer flux and protein chemistry studies, nutraceutical-treated cell samples were compared against appropriate matched controls. All data is expressed as the mean ± standard error of the mean (SEM) with the number of replicates provided for each set of studies. Differences between means are evaluated by Student's t tests for two groups.

## Results

For each nutrient compound that was tested, the concentration range chosen was based upon the concentration shown to achieve maximal barrier enhancement in other published studies, even though the epithelial model used may have differed from the LLC-PK_1_ model used here. We then tested our own concentration range that typically went at least 5-fold above and below this published concentration, in order to determine a concentration of the nutrient compound that achieved maximal barrier enhancement in our LLC-PK_1_ cell layers.

**Figure 1:**
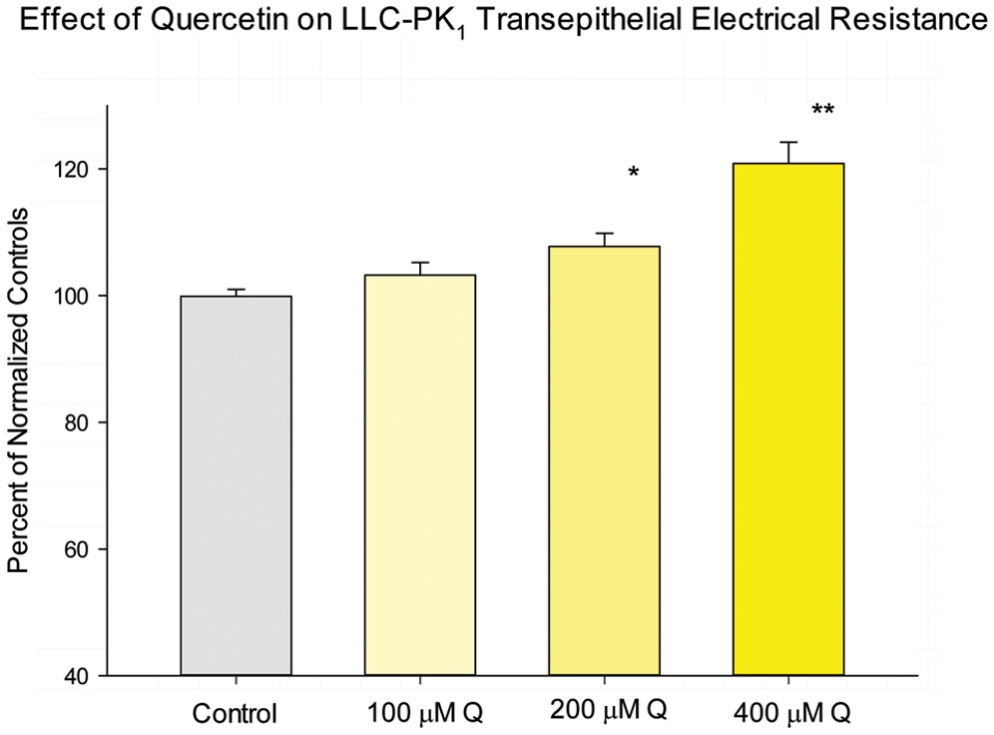
The effect of quercetin on LLC-PK_1_ transepithelial electrical resistance. LLC-PK_1_ cell layers on Millipore PCF filters were refed in control medium (apical and basal-lateral compartments) or medium containing 100, 200 or 400 µM quercetin, 48 hrs prior to electrical measurements. Data shown represents the mean ± standard error of 12 cell layers per condition. Data represents the percent of control resistance normalized for each experiment (4 experiments, 3 cell layers per experiment). * indicates P<0.02; ** indicates P<0.002 (Student's t test, one-tailed).

For each dietary compound, three concentrations were then tested, and effects of the nutraceutical on transepithelial electrical resistance (R_t_) and transepithelial D-mannitol flux (J_m_) were measured as described in Methods. Exposure times were specific for each compound (and based upon prior published results), and are specified below. In all cases, compounds were presented simultaneously to both the apical and basal-lateral cell surfaces for the times indicated. The results of the three concentrations examined are only shown for quercetin (Figure 1), but three concentrations were performed for all compounds. The concentrations reported in Tables 1 and 2 represent the concentration that achieved the maximal effect on R_t_ without any increase in J_m_. For quercetin, the concentration reported achieved a significant decrease in J_m_. For nicotine, there were no significant effects on either R_t_ or J_m_ at any concentration tested.

**Table 1:** Permeability Changes in LLC-PK_1_ Cell Layers Induced by Nutraceutical Compounds.

	Transepithelial Resistance	Transepithelial Mannitol Flux
Zinc (100 µM) (21)	34%±7% increase***	1%±4% increase (NS)
Quercetin (400 µM) (12)	42%±12% increase***	21%±5% decrease***
Indole (1 mM) (12)	24%±3% increase***	4%±4% decrease (NS)
Butyrate (1 mM) (6)	99%±12% increase***	8%±11% increase (NS)
Nicotine (10 µM) (5)	2%±1% decrease (NS)	3%±9% decrease (NS)

**Figure 2:**
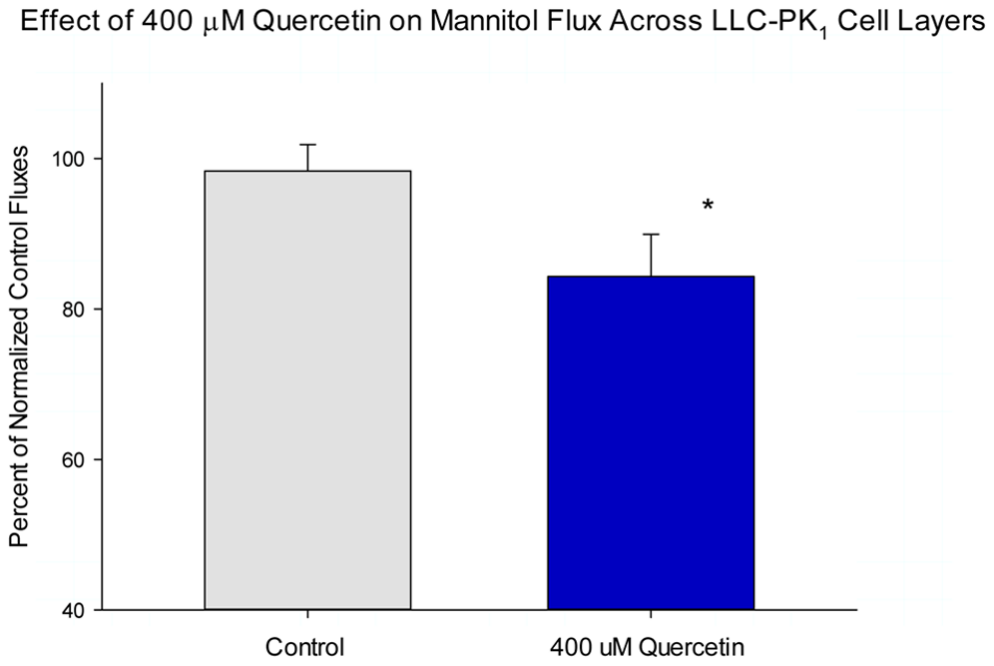
The effect of quercetin on LLC-PK_1_ transepithelial flux of ^14^C-D-mannitol. LLC-PK_1_ cell layers on Millipore PCF filters were refed in control medium (apical and basal-lateral compartments) or medium containing 400 µM quercetin, 48 hrs prior to flux studies. Data represents the percent of control flux rate normalized for each experiment (2 experiments, 3 cell layers per experiment), and is expressed as the mean ± standard error for 6 cell layers per condition. * indicates P<0.05 (Student's t test, one-tailed).

Summary of the effect of the various nutraceuticals on transepithelial electrical resistance and transepithelial mannitol flux rates across LLC-PK_1_ cell layers. Experiments were conducted as described in Figures 1 and 2. Data is shown for the optimal concentration of each nutraceutical (the concentration showing the maximal increase of resistance with (when present) the maximal decrease of mannitol flux. Data shown represents the mean ± standard error, with the n value for cell layers in parentheses.

*** indicates statistical significance to at least the P<0.005 level.

**Table 2:** Effects of Individual Nutraceuticals on LLC-PK_1_ Tight Junctional Proteins.

	% of matched control cell layers	% increase/decrease
QUERCETIN		
Claudin 1:	93%±13% (NS)	7% decrease
*Claudin 2:*	*24%±07% (P<0.005)*	76% decrease*
*Claudin 3:*	*69%±03% (NS)*	*31% decrease*
**Claudin 4:**	**145%±24% (NS)**	**45% increase**
**Claudin 5:**	**286%±39% P<0.02)**	186% increase*
**Claudin 7:**	**310%±79% P<0.05)**	209% increase*
Occludin:	115%±06% NS)	15% increase
Tricellulin:	74%±03% (NS)	26% decrease
INDOLE		
Claudin 1:	80%±13% (NS)	20% decrease
*Claudin 2:*	*50%±12% (NS)*	*50% decrease*
Claudin 3:	82%±05% (NS)	18% decrease
**Claudin 4:**	**196%±08% (NS)**	**96% increase**
**Claudin 5:**	**263%±34% (P<0.02)**	163% increase*
Claudin 7:	107%±11% (NS)	7% increase
Occludin:	6%±06% (NS)	6% increase
Tricellulin:	92%±07% (NS)	8% decrease
BUTYRATE		
**Claudin 1:**	**137%±13% P<0.05)**	37% increase*
*Claudin 2:*	*53%±05% (P<0.05)*	47% decrease*
**Claudin 3:**	**196%±10% (P<0.005)**	96% increase*
**Claudin 4:**	**285%±34% (P<0.01)**	185% increase*
**Claudin 5:**	**399%±83% (P<0.05)**	299% increase*
*Claudin 7:*	*64%±35% (NS)*	*36% decrease*
Occludin:	105%±06% (NS)	5% increase
*Tricellulin:*	*70%±11% (NS)*	*30% decrease*
NICOTINE		
Claudin 1:	99%±10% (NS)	1% decrease
Claudin 2:	92%±24% (NS)	8% decrease
Claudin 3:	104%±05% (NS)	4% increase
Claudin 4:	113%±09% (NS)	13% increase
Claudin 5:	116%±10% (NS)	10% increase
Claudin 7:	97%±03% (NS)	3% decrease
Occludin:	128%±19% (NS)	28% increase
Tricellulin:	114%±10% (NS)	14% increase
ZINC		
Claudin 1:	110%±14% (NS)	10% increase
*Claudin 2:*	*66%±17% (NS) (n = 5)*	*34% decrease*
*Claudin 3:*	*60%±29% (NS) (n = 4)*	*40% decrease*
*Claudin 4:*	*64%±17% (P<0.03)(n = 5)*	36% decrease*
**Claudin 5**:	**236%±61% (P<0.04) (n = 5)**	136% increase*
**Claudin 7:**	**158%±30% (NS)**	**58% increase**
Occludin:	85%±15% (NS)	15% decrease
Tricellulin:	111%±09% (NS)	11% increase

**Figure 3:**
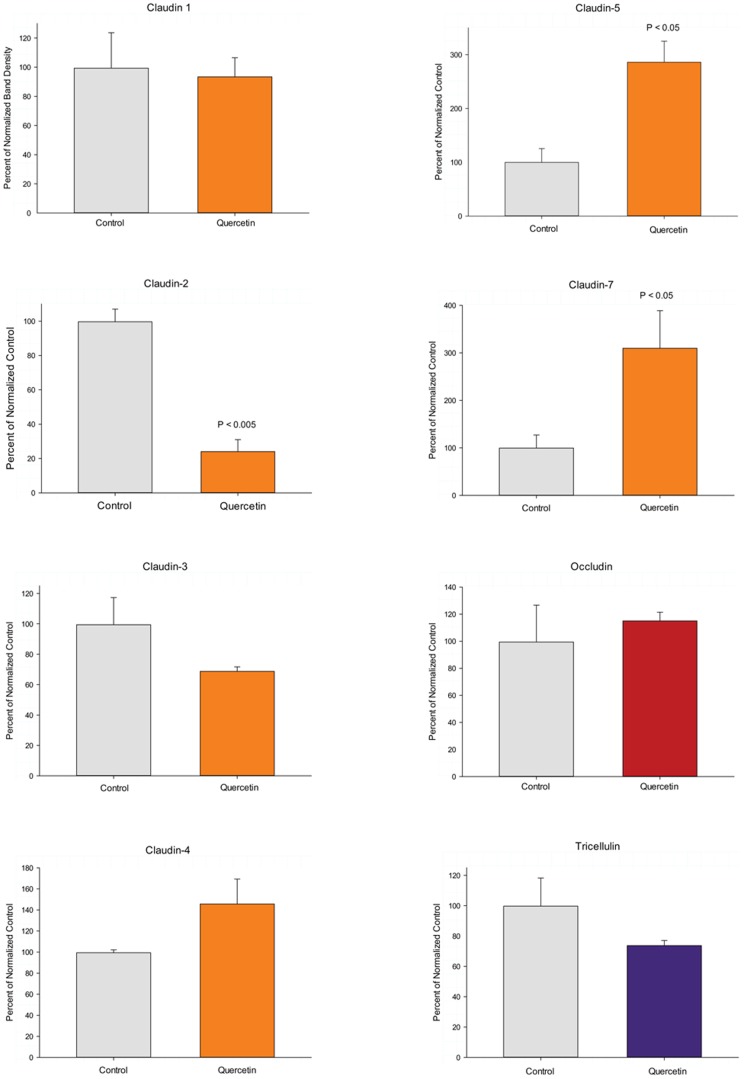
Relative changes in abundance of tight junctional proteins as a result of quercetin exposure. Post-confluent LLC-PK_1_ cell layers in Falcon 75 cm^2^ culture flasks were refed with control medium or medium containing 400 µM quercetin 48 hrs before harvesting in lysis buffer. These total cell lysates were analyzed by PAGE followed by immunoblotting as described in Methods. Immunoblots were probed with primary antisera against specific tight junctional antigens also as described. Densitometry was performed on developed blots to quantitate band densities. Three separate cultures and immunoblots were so analyzed. Results shown represent the mean ± standard error with the quercetin treatment group normalized to their respective controls. NS indicates no statistical significance (P>0.05). Where statistical significance was achieved, the P value is provided (Student's t test, one-tailed).

Summary of effects of various nutraceuticals on a panel of eight tight junctional proteins in LLC-PK_1_ cell layers. Studies were performed as described for quercetin in Figure 3. Data shown represents the mean ± standard error for an n = 3 cell layers in all cases. P values are listed for instances of statistical significance (Student's t [one tailed] to at least the P<0.05 level). NS indicates non significance. The nutraceutical concentration tested was the value shown in Table 1, the concentration providing the maximal enhancement of barrier function. Boldface signifies greater than 30% increase; italics signifies greater than 30% decrease induced by that nutraceutical in order to emphasize the various patterns specific to each nutraceutical.

The results on transepithelial permeability across LLC-PK_1_ cell layers, for the flavonoid nutraceutical, quercetin, are detailed in Figures 1 and 2. Results for all five of the nutraceuticals that we studied are summarized in Table 1. As shown in Figure 1, increasing concentrations of quercetin caused moderate, dose-dependent and statistically significant increases in R_t_. The highest quercetin concentration (400 µM) (in a 48 hr exposure) caused a 20% increase in R_t_ that was highly significant (P<0.001). When 400 µM quercetin was tested for its effect on J_m_, there was a statistically significant (15%) reduction in transepithelial mannitol leak, indicating that the barrier enhancement was to both small electrolytes and nonelectrolytes. If 15% changes in J_m_ and 20% changes in R_t_ seem only moderate enhancements, it should be considered that these nutrient compounds are attempting to ‘improve’ a barrier that is very likely in a near optimal state to begin with. A 20% *decrease* in electrolyte permeability across tight junctions is actually quite remarkable given that this is a change from the control state, not a recovery from a compromised, impaired state.

When one examines the effects of the optimal concentration (the concentration that gave the best combination of increased R_t_ and decreased [or at least unchanged] J_m_) of all five dietary nutrient compounds, it can be seen that zinc, quercetin, indole and butyrate induced significant increases in R_t_ but only quercetin caused a simultaneous statistically significant decrease in J_m_ (Table 1). Using our protocol, butyrate, indole and zinc both increased R_t_ while having no statistically significant effect on J_m_. For the case of 1 mM butyrate, this lack of an effect on J_m_ was in spite of a very dramatic increase of R_t_ (100%). Only quercetin simultaneously increased R_t_ and decreased J_m_ significantly, i.e. decreased junctional leak of both small electrolytes *and* the nonelectrolyte, mannitol. The alkaloid, nicotine, was without effect on either R_t_ or J_m_ at a concentration range of 0.1 to 10 µM, even though short circuit current was reduced by 50% at the two higher nicotine concentrations (data not shown).

The effect of the 400 µM quercetin concentration on the abundance of a panel of eight tight junctional proteins is shown in Figure 3. A highly significant 200% increase was observed for claudin-5 and a highly significant 80% *de*crease observed for claudin-2. A 200% increase was also observed for claudin-7, though not quite statistically significant (P = 0.06; the lack of statistical significance [P>0.05] was likely due to the relatively small sample size [n = 3 cell layers], and the strong effect that a small sample size has on Student t test results). No significant changes were seen regarding the amounts of occludin or claudin-1, as a result of quercetin exposure. 25% and 30% decreases in tricellulin and claudin-3 abundance respectively were observed as a result of quercetin exposure but these fell short of statistical significance perhaps for the reason mentioned above. Quercetin also caused a 45% increase in the level of claudin-4.

A summary of the effects of all five of the compounds tested is shown in Table 2, for the same concentrations used in Table 1. None of the five nutraceuticals had any effect on occludin, and only butyrate had any significant effect on claudin-1 (a 37% increase). There were also no significant effects on tricellulin, although the 30% decreases in tricellulin in the presence of quercetin and butyrate were close to statistical significance. Nicotine, which had no significant effect on R_t_ or J_m_, also had no significant effects on overall abundance of any of the tight junction proteins analyzed. For the four nutraceuticals that did have effects on either R_t_ and/or J_m_, note that the overall pattern of effects of each nutraceutical on the various tight junctional proteins were unique to each nutrient compound.

**Figure 4:**
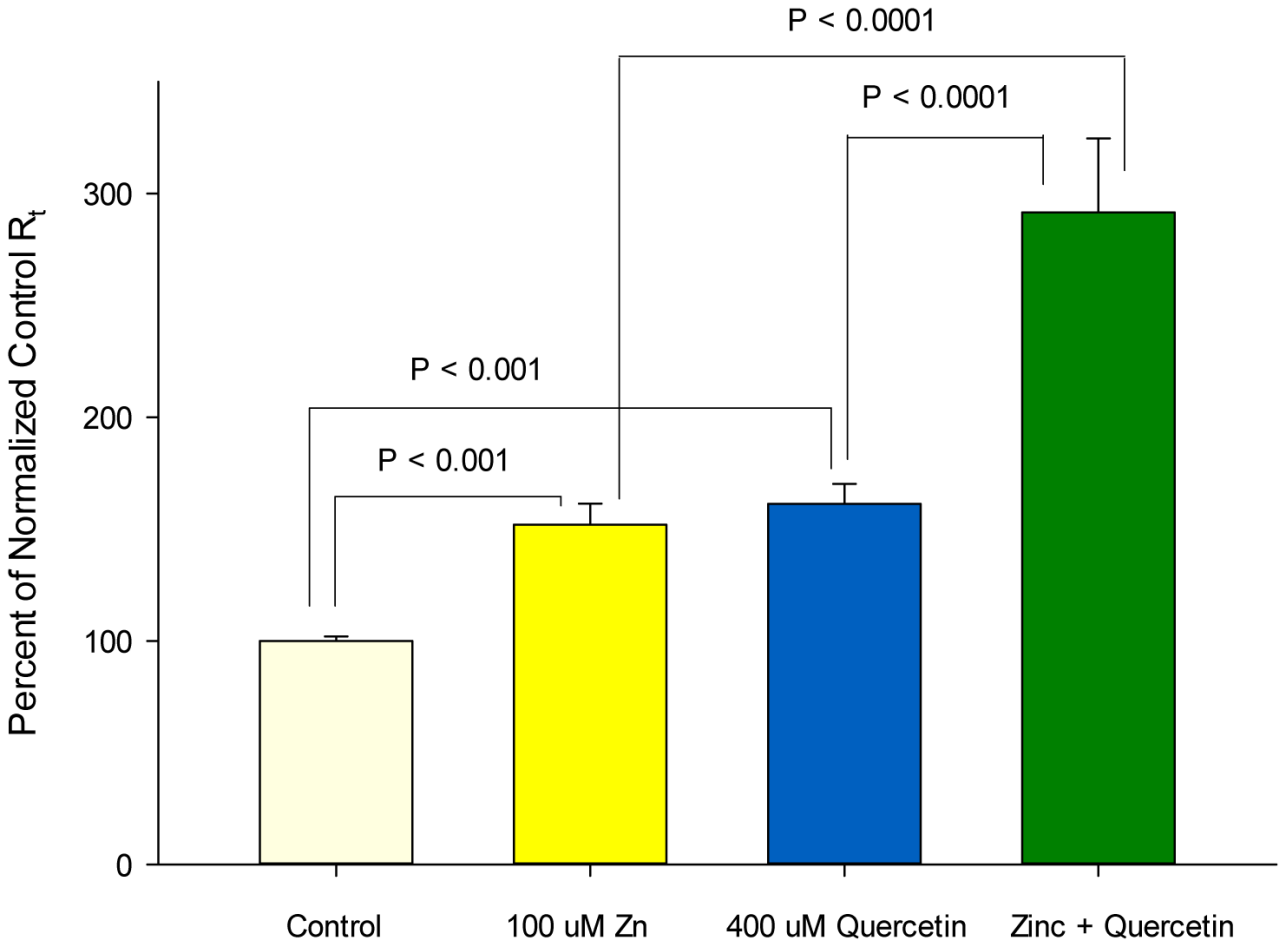
Additive effect of a zinc/quercetin combination on LLC-PK_1_ transepithelial electrical resistance relative to either agent alone. Cell layers were exposed to zinc (100 uM), quercetin (400 uM) or their combination for 48 hours. Data represents the percent of control resistance normalized for each experiment (3 experiments, 4 cell layers per experiment), and is expressed as the mean ± standard error for 12 cell layers per condition. P values (Student's t test, one-tailed) are indicated for statistical comparisons of the various conditions.

**Figure 5:**
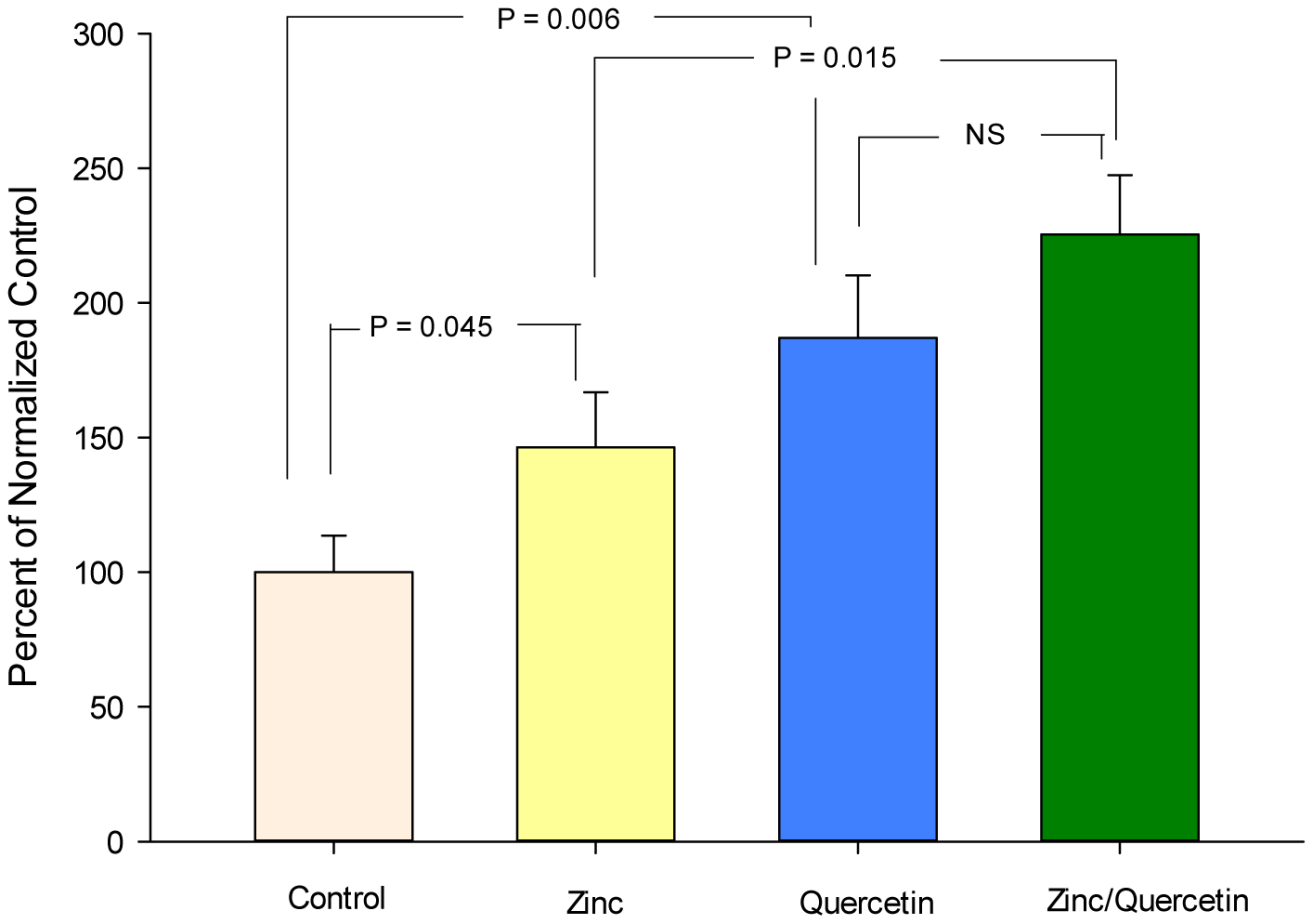
Effect of a zinc/quercetin combination on the tight junctional protein, claudin-7. Data represent the mean ± standard error of 5 cell layers. The zinc and quercetin conditions individually were statistically different from the control. The combination was also statistically different from the control (P<0.01) as well as zinc alone (P = 0.015). The combination induced a 50% greater increase in claudin-7 than quercetin alone, but the difference did not reach statistical significance (P = 0.13). P values derive from the Student's t test, one-tailed. Concentrations of the nutraceuticals are the same as in Figure 4.

As shown in Figure 4, the combination of the optimal concentrations of zinc and quercetin resulted in a synergistic enhancement of R_t_. Although this powerful effect was observed on R_t_, the combination failed to produce a decrease in J_m_. The combination moreover did not exhibit any additive or synergistic effects on specific tight junctional proteins, with the possible exception of claudin-7 (Figure 5).

## Discussion

In the tight junctional complex, the transmembrane tight junctional proteins - most notably occludin, tricellulin and the 26 different claudins - constitute the physical barrier to paracellular diffusion across the epithelium. This study determined that a group of four very different dietary compounds (that are each capable of enhancing the barrier properties of the epithelial cell layer) each produce unique, distinct effects on the amount of specific barrier proteins in the TJ complex. In addition, each had unique effects concerning the nature of the enhancement of barrier function. These differences among the effects of the agents sets the stage for future combinational use of these agents to possibly generate still different effects (and possibly effects of greater clinical benefit) on the tight junctional complex. These combinations may produce additive or even synergistic enhancement of the barrier function of specific epithelial cell layers. We have previously shown that a single dietary compound has unique effects on tight junctional complexes depending upon the nature of the epithelial cell layer under study [22]. Now we can add to that finding by saying that the effects of different compounds on the same cell layer are unique to the compound, even though as a group they all act to enhance the cell layer barrier.

In the group of zinc, quercetin, indole, butyrate and nicotine, all of the agents except nicotine acted on the LLC-PK_1_ cell layer to reduce its passive leakiness. However, only quercetin (at 400 µM) acted to not only increase R_t_ but also decrease J_m_. Thus, in response to quercetin, passive leak to electrolytes as well as small nonelectrolytes was reduced. Zinc, buytyrate and indole increased R_t_, but did not affect J_m_. Butyrate's effect on R_t_ was over 3× the effect of zinc and indole. The precise type of barrier enhancement is therefore different from compound to compound.

Given the unique pattern of effects by each agent on the panel of TJ proteins under study (Figure 3 and Table 2); it is really not surprising that each would enhance TJ barrier function differently. These differences moreover suggest that certain combinations of these compounds might induce a still further modified TJ complex that may possess yet higher R_t_ values (than for the compounds individually) and/or lower J_m_ values. This possibility was tested in the experiments reported in Figure 4. In those studies, the combination of 100 µM zinc and 400 µM quercetin induced an increase in R_t_ that was greater than the sum of the induced increases by either compound alone. This combination, however, failed to reduce J_m_ (data not shown), as was seen with 400 µM quercetin alone (Figure 2). This may simply highlight the overall complexity of epithelial barrier regulation in its totality (unstirred layers, tight junctions, cell viability, etc), as well as the fact that the nutraceuticals in question likely have manifold effects on epithelial cells quite apart from actions on the TJ complexes. Although the zinc/quercetin combination had a dramatic effect on R_t_, it was noteworthy that there was no observed additive, synergistic or de-novo effects on TJ proteins with the possible exception of claudin-7. In this regard it should be highlighted that in this study we have looked only at up and downregulation of the various TJ proteins (in total cell lysates). We have left unaddressed possible nutraceutical-induced changes in TJ protein subcellular localization as well as phosphorylation state, both of which likely play roles in barrier function

Concerning the actions of the various dietary compounds on the panel of TJ proteins under study, it is noteworthy that none of the compounds improving barrier function significantly altered levels of occludin (Table 2). Claudin-1 was not significantly changed by any nutraceutical except butyrate. Claudine-2 was reduced in every instance where a significant increase in R_t_ was observed. Claudin-5 was significantly and dramatically increased by every nutraceutical that augmented barrier function. Nicotine, which exerted no significant effects on barrier function (Table 1), was likewise without significant effect on the abundance of any of the tight junction proteins analyzed (Table 2). The most noteworthy effect of the different nutraceuticals on tight junctional proteins was, in our opinion, the overall pattern of effects. In Table 2 we have highlighted in green, induced decreases of proteins that were greater than 40%. Yellow highlighting indicates induced increases that were greater than 40%. The highlighting serves to thus emphasize the unique actions of each nutraceutical on the panel of tight junctional proteins that were analyzed, even though butyrate, zinc, indole and quercetin all acted similarly to improve barrier function.

As alluded to above, the situation regarding nicotine is distinct from the other compounds. As shown in Table 1, nicotine (at neither 0.1, 1 nor 10 µM) had no effect on either R_t_ or J_m_. This was true even though dramatic 50% reductions of I_scc_ were consistently observed at the 10 µM concentration, proof of biological activity of the compound at that concentration [data not shown]). In keeping with this lack of action by nicotine on barrier function, there were no significant effects of nicotine on any of the TJ proteins that were analyzed (Table 2).

Before concluding, it may be worthwhile to point out the value of research on TJ composition and permeability by simple and commonplace dietary compounds, as opposed to more ‘high powered’ efforts involving oligonucleotides, small inhibitory RNAs or micro RNAs to achieve “designer” TJ complexes. As was pointed out above, the TJ complex consists of approximately 30 (known) barrier TJ proteins (in addition to the intracellular tight junctional-associated proteins). It is quite likely that these proteins function in zipper-like fashion through a variety of homotypic and heterotypic interactions [29]. If one has a complex of e.g. 30 constituent proteins which are capable of homotypic and heterotypic interactions with each other, one has a potential set of 30 factorial (or 1×10^28^) potential interaction pairings. If one seeks to obtain a “better TJ complex” by rational design directed at simply upregulation or downregulation of individual proteins (tabling for the moment the reality that these individual proteins can be modified by phosphorylation state as well), one is confronted by a myriad of possibilities as one searches for the perfect, optimal state. On the other hand one could utilize an approach based on modulators vetted by evolution, i.e. the nutraceuticals. Our cells and tissues have been in contact with substances such as zinc, indole and quercetin for millennia. If these substances have a positive effect on epithelial barriers in our tissues it is an adaptational process that has been worked out for eons. Moreover, if the actions of one of these substances are salutary for an epithelial layer's barrier function, this action likely evolved without detriment to other cells and organ systems by the very nature of it being an adaptational advantage to the entire organism. It is unlikely that our GI tract would evolve to have a salutary response to quercetin if quercetin was also causing, e.g. leakage in the blood brain barrier or diminished capacity for paracellular magnesium reabsorption in the kidney. The overall evolutionary advantage *to the organism* would not be there. The same cannot be said e.g. for a potential new siRNA that would be discovered to have gastrointestinal barrier enhancing effects through in vitro studies at the lab bench.

In the near future, use of these nutrient agents will likely advance in various clinical situations characterized by barrier compromise (e.g. inflammatory bowel disease [30], [31] or partial failure of the respiratory, renal and GL barriers in multi-organ failure [9], [15]). In such scenarios it may prove quite useful that the barrier effects of the dietary compounds will be unique to the individual epithelial cell layer, and also to the specific compound in use. In potential future clinical use, one can't e.g. expect zinc alone to be efficacious for every epithelial tissue, nor can one expect that quercetin and butyrate will force changes in transepithelial permeability to the same set of substances. Both of these scenarios then highlight the potential value in combinations of agents for the purpose of barrier enhancement to wider ranges of solutes, and also a wider range of barriers (epithelial and endothelial tissues) capable of being enhanced together, at the same time. These combinations will need to be carefully researched to find clinically optimal formulations. Occurrence of combinations of these compounds in specific foods could serve as a useful guide in this regard.

## Conclusions

The fact that indole, quercetin, butyrate and zinc all improve epithelial barrier function but do so with quite different patterns of effects on a panel of eight different tight junctional proteins indicates that: 1) tight junctional complexes can have their barrier function enhanced by a variety of pathways with different structural and permeability outcomes; 2) these different outcomes being produced by different nutraceuticals opens the door to the possibility of using combinations of nutraceuticals to achieve additive or even synergistic effects regarding enhancement of barrier function.
